# Unraveling the Genetic Basis of Combined Deafness and Male Infertility Phenotypes through High‐Throughput Sequencing in a Unique Cohort from South India

**DOI:** 10.1002/ggn2.202300206

**Published:** 2024-05-08

**Authors:** Jeffrey Justin Margret, Chandru Jayasankaran, Pavithra Amritkumar, Hela Azaiez, C. R. Srikumari Srisailapathy

**Affiliations:** ^1^ Department of Genetics Dr. ALM Post Graduate Institute of Basic Medical Sciences University of Madras Taramani Campus Chennai 600 113 India; ^2^ Department of Pediatrics Louisiana State University Health Sciences Center Shreveport LA 71103 USA; ^3^ Department of Personalized Health Care Roche Products India Pvt., Ltd. Bengaluru Karnataka 560 025 India; ^4^ Meenakshi Academy of Higher Education and Research (MAHER) Chennai 600 078 India; ^5^ Department of Otolaryngology Carver College of Medicine University of Iowa Iowa City Iowa 52242 USA

**Keywords:** deafness and male infertility syndrome, exome sequencing, *GJB2*, hearing loss, male infertility

## Abstract

The co‐occurrence of sensorineural hearing loss and male infertility has been reported in several instances, suggesting potential shared genetic underpinnings. One such example is the contiguous gene deletion of *CATSPER2* and *STRC* genes, previously associated with deafness‐infertility syndrome (DIS) in males. Fifteen males with both hearing loss and infertility from southern India after exclusion for the DIS contiguous gene deletion and the *FOXI1* gene mutations are subjected to exome sequencing. This resolves the genetic etiology in four probands for both the phenotypes; In the remaining 11 probands, two each conclusively accounted for deafness and male infertility etiologies. Genetic heterogeneity is well reflected in both phenotypes. Four recessive (*TRIOBP, SLC26A4, GJB2, COL4A3*) and one dominant (*SOX10*) for the deafness; six recessive genes (*LRGUK, DNAH9, ARMC4, DNAH2, RSPH6A*, and *ACE*) for male infertility can be conclusively ascribed. *LRGUK* and *RSPH6A* genes are implicated earlier only in mice models, while the *ARMC4* gene is implicated in chronic destructive airway diseases due to primary ciliary dyskinesia. This study would be the first to document the role of these genes in the male infertility phenotype in humans. The result suggests that deafness and infertility are independent events and do not segregate together among the probands.

## Graphical abstract

A comprehensive analysis of the genetic makeup of a unique cohort of males with hearing impairment and infertility uncovers a wide range of gene variations spanning seven chromosomes, highlighting significant genetic heterogeneity within this disorder. The analysis states that hearing impairment and infertility in these individuals are independent events and are not associated with a syndrome‐related disorder.

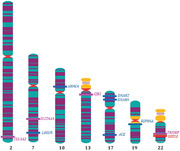

## 1 Introduction

Hearing loss (HL) is the most frequent sensory disorder. Hearing is a complex and sophisticated mechanism involving a cascade of cell types and cellular and molecular architectures and mechanisms.^[^ ^1^ ^]^ Over 150 genes have been associated with genetic HL.^[^ ^2^ ^]^ Hereditary HL may occur as an isolated condition in nonsyndromic HL or as part of a syndrome with other associated manifestations. Syndromic HL is phenotypically and genetically complex with over 400 genetic syndromes identified to date.^[^ ^3^ ^]^

Infertility is yet another complex disorder with a spectrum of phenotypes. It affects 15% of couples worldwide,^[^ ^4^ ^]^ with 40% of those due to male infertility (MI).^[^ ^5^ ^]^ In India, between 12 and 18 million, couples are diagnosed with infertility every year with half of the cases due to MI. Approximately 30% of MI cases have a genetic etiology^[^ ^6^ ^]^ and 25–30% are idiopathic and may be linked to unknown genetic defects.^[^ ^7^ ^]^ Although 2000 genes have been associated with spermatogenesis,^[^ ^8^ ^]^ only 0.01% of these genes have been evaluated in infertile men in genetic labs worldwide.^[^ ^9^ ^]^ Environmental factors such as occupational exposure to toxins, lifestyle (smoking, alcohol consumption, drugs, obesity, psychological stress), and chronic health issues (testicular torsion, epididymitis, urethritis, varicocele) also play a role in MI.^[^ ^10^ ^]^

HL and MI have been associated individually with many other syndromes. Deafness and male infertility syndrome (DIS) is characterized by both HL and MI. It is caused due to a contiguous deletion of the *STRC* and *CATSPER2* genes on 15q15.3.^[^ ^11^ ^]^ Stereocilin, a structural protein, is encoded by the *STRC* gene and is found in the stereocilia of the outer hair cells within the inner ear. It plays a crucial role in the process of mechanoreception, as it converts sound waves into electrical signals.^[^ ^12^ ^]^ *CATSPER2* regulates the influx of positively charged calcium ions into sperm cells, enhancing their motility.^[^ ^11^ ^]^ The severity of HL in this syndrome is typically mild to moderate but cases of profound HL were also reported.^[^ ^13^ ^]^

The prevalence of DIS is not well‐documented. A study conducted in the USA suggests that around 1 in 40000 individuals are born with a homozygous deletion of the *CATSPER2/STRC* region and estimated a carrier frequency of 1.09%.^[^ ^13^ ^]^ A recent study conducted in Japan analyzed 9956 HL patients using NGS and CNV analysis and showed that 2.32% (231) of the patients had a homozygous deletion in the *STRC* gene. Out of these 231 patients, 140 were randomly selected for MLPA, and it was found that 108 individuals (52 males) had a deletion in the *CKMT1B‐STRC*‐*CATSPER2* region. Based on these findings, the estimated frequency of DIS in the Japanese population is ≈2%.^[^ ^14^ ^]^ The prevalence of DIS in the Indian population remains unexplored as only one case has been reported so far.^[^ ^15^ ^]^

The *FOXI1* gene plays a major role in the early development of the vestibule and cochlea in the inner ear and is a potential transcriptional activator of the *SLC26A4* gene.^[^ ^16^, ^17^ ^]^ Further, it regulates proton secretion in narrow and clear cells of the epididymis and the storage of spermatozoa. Deficiency in the FOXI1 protein results in immature sperm and leads to male infertility in mice.^[^ ^18^ ^]^

In this study, we aim to investigate the genetic etiology of comorbidity of HL and MI in a unique cohort of individuals from southern India who were negative for *STRC‐CATSPER2* contiguous gene deletion and *FOXI1* gene mutations.

## 2 Results

An average of 72 500 exonic and 2000 splice‐site variants were identified in each proband before filtering. Variants with greater than 1% minor allele frequencies were filtered out.

Exome analysis resolved the genetic etiology of HL in six families (DIS‐2, DIS‐3, DIS‐9, DIS‐10, DIS‐12, and DIS‐15) (**Table** **1**) whereas plausible causative genes for MI were identified in six families (DIS‐2, DIS‐8, DIS‐9, DIS‐12, DIS‐13, and DIS‐15) (**Table** **2**).

**Table 1:** Variants implicated for the hearing loss phenotype in the study cohort.

S. No	Family ID	Gene	HGVS	Variant Type	Zygosity	gnomAD (%)	In silico predictions				Segregation Analysis^#^	ACMG Attributes	Classification	dbSNP	Reference
							SIFT	PP	MT	ConSurf					Pubmed/ Clinvar ID
1	DIS 2	*TRIOBP*	NM_001039141.3: c.2320C>T p.(Arg774Ter)	NS	Homo	0. 000401	D	PoD	DC	5	Yes	PVS1, PM2, PM3_supp, PP1	Pathogenic	rs529745904	27848944
2	DIS 3	*SLC26A4*	NM_000441.2: c.269C>T p.(Ser90Leu)	MS	Homo	0. 00199	D	PrD	DC	8	Yes	PM2_supp, PM3_very strong, PP1_strong, PP3,	Likely Pathogenic	rs370588279	19287372
3	DIS 9	*GJB2*	NM_004004.6: c.71G>A p.(Trp24Ter)	NS	Homo	0. 0584	D	PrD	DC	9	Yes	PVS1, PM3_very strong, PP1_strong	Pathogenic	rs104894396	9139825
4	DIS 10	*SOX10*	NM_006941.4: c.1315_1329del p.(Ile439_Ser443del)	IFD	Het	A	‐	‐	DC	‐	ND	PM4, PM2, PP4	VUS	**Novel** rs1569167515	ClinVar ID: 620636
5	DIS 12	*GJB2*	NM_004004.6: c.104T>G p. (Ile35Ser)	MS	Homo	0. 000797	D	PrD	DC	8	Yes	PS4, PP3, PM2, PM3_very strong, PP1_strong	Pathogenic	rs756467247	18941476
6	DIS 15	*COL4A3*	NM_000091.5: c.387+1G>A	SS	Homo	A	‐	‐	DC	‐	Yes	PVS1, PM2	Likely Pathogenic	**Novel** rs1574674108	ClinVar ID: 812587

ACMG, American College of Medical Genetics and Genomics variant classification^[^ ^75^ ^]^; NS, Nonsense; MS, Missense; IFD, Inframe Deletion; SS, Splice site; Homo, Homozygous; Het, Heterozygous; PP, PolyPhen2; MT, MutationTaster; D, Damaging; PrD, Probably Damaging; PoD, Possibly Damaging; DC, Disease Causing; A, Absent; ND, Not Done. gnomAD, Minor Allele Frequency of the variant; Consurf scores, 1 is variable and 9 is conserved; #Segregation done with the available family members.

**Table 2:** Variants implicated for male infertility phenotype in the study cohort.

S. No	Family ID	Gene	HGVS	Variant Type	Zygosity	gnomAD (%)	In silico predictions				Segregation Analysis^#^	ACMG Attributes	Classification	dbSNP	Reference	
							SIFT	PP	MT	ConSurf					Pubmed ID*	Clinvar ID
1	DIS 2	*LRGUK*	NM_144648.3: c.890G>T p.(Gly297Val)	MS	Homo	0.3025	D	PrD	DC	9	Yes	PM1, PP1, PP3	VUS	rs79217401	25781171	‐
2	DIS 8	*DNAH9*	NM_001372.4: c.1405G>A p.(Gly469Ser)	MS	Comp Het	0.007954	D	PrD	DC	8	Yes	PM2, PM3, PP3	VUS	rs559139037	15750039	‐
3			NM_001372.4: c.4550G>A p.(Arg1517Gln)	MS		0.1033	D	PrD	DC	7	Yes	PM3, PP3, BS1	VUS	rs139715944	15750039	‐
4	DIS 9	*ARMC4*	NM_018076: c.2097+1G>A	SS	Comp Het	0.003	‐	‐	DC	‐	Yes	PVS1, PM2, PM3, PP3	Pathogenic	**Novel** rs149368374	23849778	654954
5			NM_018076: c.2765G>A p.(Gly922Glu)	MS		0.000403	D	PrD	DC	8	Yes	PM2, PM3, PP3	VUS	**Novel** rs1564439559	23849778	812720
6	DIS 12	*DNAH2*	NM_020877.4): c.2479C>T p.(Arg827Trp)	MS	Homo	0.0012	D	PrD	DC	3	Yes	PM2, PP2	VUS	rs760771496	30811583	‐
7	DIS 13	*RSPH6A*	NM_030785.4): c.1939G>A p.(Gly647Ser)	MS	Homo	0.002584	D	PrD	DC	9	Yes	PM2, PP3, BS1	VUS	rs770885407	30185526; 30239614	‐
8	DIS 15	*ACE*	NM_000789.4): c.2912G>A p.(Arg971Gln)	MS	Homo	0.00799	D	PrD	DC	8	Yes	PVS1, PM2, PP3	Pathogenic	rs554004241	9482924	‐

ACMG, American College of Medical Genetics and Genomics variant classification^[^ ^76^ ^]^; MS, Missense; SS, Spice site; NA, Not Applicable; Homo, Homozygous; Comp Het, Compound Heterozygous; PP, PolyPhen2; MT, MutationTaster; D, Damaging; PrD, Probably Damaging; DC, Disease Causing; A, Absent. gnomAD, Minor Allele Frequency of the variant; Consurf scores, 1 is variable and 9 is conserved; #Segregation done with the available family members; *Publication related to the functional analysis of the whole gene.

### 2.1 Genetic Etiology Resolved for Both HL and MI

**Figure 1:**
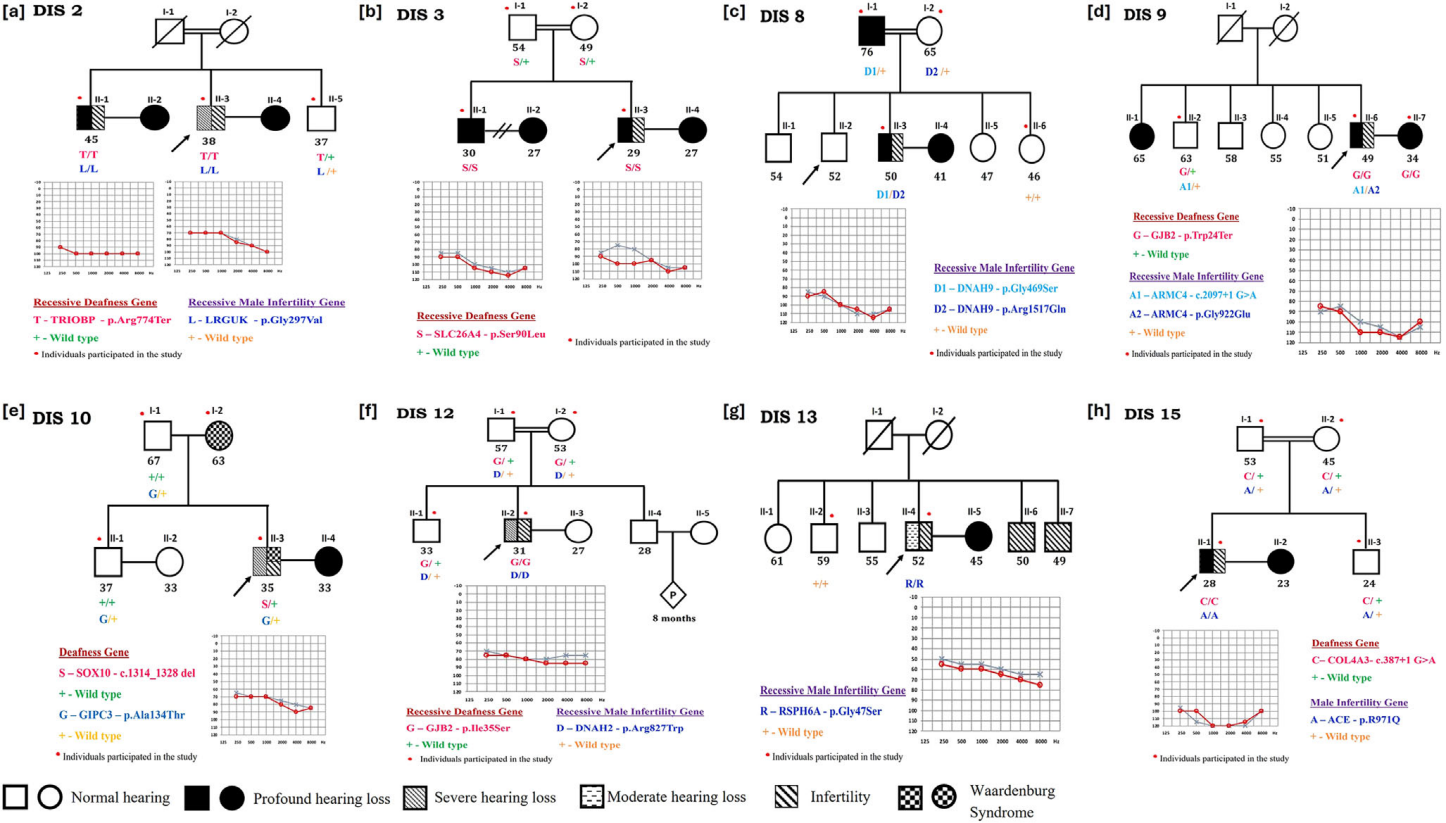
Segregation pattern of the variants identified in the auditory and/or male infertility genes among the families a) DIS2; b) DIS3; c) DIS8; d) DIS9; e) DIS10; f) DIS12; g) DIS13, and h) DIS15.Individuals participating in the study are indicated with a red dot.

In proband DIS‐2 (II‐3), we identified homozygous variants in the *TRIOBP*, (NM_001039141.3:c.2320C>T: p.[Arg774Ter]) and *LRGUK*, (NM_144648.3:c.890G>T: p.[Gly297Val]) genes for HL and MI, respectively. The *TRIOBP* variant is ultra‐rare (MAF <0.0004% in gnomAD) and predicted to be deleterious. The glycine at 297 position in *LRGUK* resides in the LRR 9 (Leucine‐rich repeat) region. It is highly conserved and its change to valine is predicted deleterious. These variants were also observed in a homozygous state in his affected brother (II‐1) and a heterozygous state in his unaffected younger brother (II‐5) (**Figure** **1**a).

Proband DIS‐9 (II‐6) was homozygous for a known pathogenic variant (NM_004004.6:c.71G>A: p.[Trp24Ter]) in the *GJB2* gene. Exome analysis revealed a novel splice‐site variant (NM_018076:c.2097+1G>A) and an ultra‐rare missense variant (NM_018076:c.2765G>A: p.[Gly922Glu]) in MI‐associated gene, *ARMC4*. The glycine at the 922 position is conserved and resides in the ARM domain. Both variants are predicted to be deleterious and are absent in all population databases. The splice site donor loss variant with CADD and SpliceAI scores of 33 and 0.99 respectively, affects the mRNA translation. The normal‐hearing unmarried elder brother (II‐1) carries only the splice‐site variant (Figure 1d).

Hearing loss in proband DIS‐12 (II‐2) was due to a rare (MAF< 0007974% in gnomAD) pathogenic variant (NM_004004.6:c.104T>G: p.[Ile35Ser]) in *GJB2*. A homozygous missense variant, (NM_020877.4: c.2479C>T: p.[Arg827Trp]) in *DNAH2* was identified as a candidate variant for his infertility. This variant is predicted to be deleterious and has a low MAF (0.00119% in gnomAD). Both the parents (I‐1 and I‐2) and normal‐hearing unmarried elder brother (II‐1) were carriers for these variants (Figure 1f).

In proband DIS‐15 (II‐1), we identified a homozygous splice‐site variant (NM_000091.5: c.387+1G>A) in the *COL4A3* gene as causative for HL. This novel donor loss variant has a CADD score of 33 and SpliceAI as 0.88 typically leads to a loss of protein function by affecting the mRNA translation. Both parents and unaffected sibling were heterozygotes for this variant. A missense variant (NM_000789.4: c.2912G>A: p.[Arg971Gln]) in the *ACE* gene was identified for MI. Arginine at position 971 is conserved and lies in the Peptidase M2 domain of the ACE protein. This variant is rare (0.008% MAF in gnomAD) and predicted to be deleterious. Segregation analysis revealed that this variant was heterozygous in both the parents (I‐1 and I‐2) and the unmarried younger brother (II‐3) (Figure 1h).

### 2.2 Genetic Etiology for HL Identified But Etiology for MI Elusive

A homozygous missense variant (NM_000441.2:c.269C>T: p.[Ser90Leu]) in exon 3 of *SLC26A4* was identified in proband DIS‐3 (II‐3). Serine at position 90 is conserved and resides in the sulfate transporter domain of the SLC26A4 protein. This variant is predicted to be deleterious with MAF 0.002% in gnomAD. This variant was observed as homozygous in his hearing‐impaired brother (II‐1) and heterozygous in his normal hearing parents (II‐1 & II‐2). The audiological evaluation of the proband showed profound SNHL in the right ear and severe to profound SNHL in the left ear, and bilateral profound SNHL in his elder brother (Figure 1b).

A novel heterozygous 13 bp deletion (NM_006941.4:c.1315_1329del: p.[Ile439_Ser443del]) in exon 5 of *SOX10* gene was observed in proband DIS‐10 (II‐3). Both the proband and his mother (I‐2) showed the features of Waardenburg syndrome (WS) with characteristic iris heterochromia; however, no depigmentation was seen in hair or skin. Audiological evaluation of the proband showed bilateral severe SNHL whereas his mother was normal hearing. His normal‐hearing father (I‐1) and brother (II‐1) did not carry this deletion. Blood sample for the mother was unavailable (Figure 1e).

### 2.3 Genetic Etiology for MI Identified But Etiology for HL Elusive

Two compound heterozygous variants, (NM_001372.4:c.1405G>A: p.[Gly469Ser]); (NM_001372.4:c.4550G>A: p.[Arg1517Gln]) in *DNAH9* gene were identified in proband DIS‐8 (II‐3). The amino acids Gly469 and Arg1517 are conserved and lie in the DHC N1 and DHC N2 (dynein heavy chain, N‐terminal) regions, respectively. Upon segregation analysis, p.(Gly469Ser) was inherited from the father (I‐1) and p.(Arg1517Gln) from his mother (I‐2) (Figure 1c).

A homozygous missense variant (NM_030785.4: c.1939G>A: p.[Gly647Ser]) in exon 6 of the *RSPH6A* gene was identified in proband DIS‐13 (II‐4). Glycine at position 647 resides in the radial spoke head‐like family protein region of RSPH6A protein and is highly conserved. This rare variant (MAF 0.0025% in gnomAD) is predicted to be deleterious. Proband's unaffected elder brother (II‐2) did not carry this variant. However, the proband's two younger brothers (II‐6 & II‐7) who were infertile were unavailable for genetic investigation (Figure 1g).

## 3 Discussion

Both hearing loss and male infertility are genetically heterogeneous disorders, and this extreme heterogeneity makes genetic diagnosis exceedingly difficult. The contiguous gene deletion of *STRC*‐*CATSPER2* causes both HL and MI.^[^ ^11^ ^]^ Similarly, two other genes *FOXI1* ^[^ ^19^ ^]^ and the *CDC14A* gene^[^ ^20^ ^]^ are known to cause both conditions. This study comprehensively investigated a unique cohort of males with HL and MI from southern India. Although no genes were identified that simultaneously explain both HL and MI, the genetic heterogeneity is well reflected in both phenotypes with five genes implicated in HL and six in MI (Figure S2, Supporting Information).

Five genes implicated in HL were identified, four of which were recessive (*TRIOBP, SLC26A4, GJB2, COL4A3*) and one was dominant (*SOX10*). Six variants (two missense, two nonsense, one splice site, and one inframe deletion) were identified in these five genes, including two novel variants (Table 1).

*TRIOBP* encodes TRIO and filamentous‐actin‐binding protein, which co‐localizes with F‐actin along the length of the stereocilia^[^ ^21^ ^]^ and plays a vital role in rootlet formation. Variants in the *TRIOBP* gene cause profound prelingual nonsyndromic HL, associated with DFNB28.^[^ ^22^ ^]^ We observed a nonsense variant p.(Arg774Ter) in exon 7 in the DIS 2 family. The repeat motifs R1 and R2 in exon 7 of TRIOBP‐5 are vital for the actin‐binding.^[^ ^23^ ^]^ Most of the pathogenic variants reported are localized in exon 7 and affect isoforms TRIOBP‐5 and TRIOBP‐4.^[^ ^21^, ^24^, ^25^, ^26^, ^27^, ^28^, ^29^ ^]^ Although *TRIOBP* gene variants are not a common cause for HL, exon 7 may be considered a hotspot region for future genetic screening of HL.

*SLC26A4* encodes pendrin which is expressed in the inner ear, kidney, and thyroid gland. It regulates anions such as Cl^−^, HCO3^−^, and I^−^ depending on the sites of its expression.^[^ ^30^ ^]^ In the inner ear, it is expressed in the apical membrane of transitional cells (saccule, utricle, and ampullae), epithelium of the endolymphatic sac and duct, and in a multitude of cell types in the cochlea.^[^ ^31^ ^]^ Decreased bicarbonate secretions can reduce fluid resorption in the endolymphatic sac resulting in HL and cochlear enlargement.^[^ ^32^ ^]^ Variants in the pendrin gene lead to autosomal recessive non‐syndromic HL (DFNB4) with enlarged vestibular aqueduct (EVA). In the DIS 3 family, we observed a homozygous pathogenic missense variant p.[Ser90Leu] in both brothers with HL. Hu et al.^[^ ^33^ ^]^ reported the association of this variant in patients with NSHL and bilateral EVA. The clinical assessment was not possible due to a lack of consent from this family. The founder effect of this variant was reported in the Pakistani HL population with a frequency of 18%.^[^ ^34^, ^35^ ^]^ The prevalence of *SLC26A4* gene variants in south India was recently reported as 3.4% in one of our previous studies.^[^ ^36^ ^]^

Connexins forming gap junctions facilitate the recycling of potassium ions between the fluids of the inner ear by regulating the cellular ion exchanges.^[^ ^37^ ^]^ The absence of potassium ions recycling impairs hair cells's ability to produce an action potential in response to sound. The frequency of the *GJB2* gene variants in the Indian population ranges from 9.90%^[^ ^38^ ^]^ to 40%.^[^ ^39^ ^]^ Two pathogenic variants p.[Trp24Ter] and p.[Ile35Ser] were observed in this study. Due to the high frequency of p.[Trp24Ter] variant, it was suggested that this may be a founder mutation in the south Indian population.^[^ ^40^, ^41^, ^42^ ^]^ The p.[Ile35Ser] is rare and previous studies have shown that it impairs trafficking of the protein to the plasma membrane.^[^ ^43^ ^]^

*COL4A3* gene, part of the type IV collagen group, encodes the primary structural protein in the basement membrane. *COL4A3* encodes collagen IV alpha 3 chain^[^ ^44^ ^]^ and is expressed in the basement membranes of the inner ear, kidney, and eye.^[^ ^45^ ^]^ Mutations in these genes are associated with the autosomal recessive form of Alport syndrome. Alport syndrome is characterized by proteinuria, hematuria, progressive renal failure, and sensorineural hearing loss or ocular abnormalities.^[^ ^46^ ^]^ In our study, we identified a novel homozygous splice site variant in proband DIS‐15 suggesting a diagnosis of Alport syndrome. However, a clinical examination was not possible as the family was untraceable for a follow‐up study. While the proband's parents and younger brother carry this variant, none of them have HL. Prior studies report that heterozygous carriers have mild HL without any renal abnormalities.^[^ ^47^ ^]^

*SOX10* gene is a member of the SOX family of transcription factors and^[^ ^48^ ^]^ is a key regulator of the development of the neural crest, enteric ganglia, and melanocytes. It also promotes the development of the peripheral nervous system and embryonic neural cells.^[^ ^49^ ^]^ Mutations in *SOX10* cause Waardenburg syndrome and account for 15% of type II and 45–55% of type IV cases.^[^ ^50^, ^51^ ^]^ WS is both clinically and genetically a heterogeneous condition, which results from melanocyte deficiency of the skin and the absence of stria vascularis in the cochlea.^[^ ^52^ ^]^ It is characterized by depigmented patches of the hair and skin, heterochromia irides, or vivid blue eyes along with sensorineural hearing loss. The proband DIS‐10 with *SOX10* deletion presents with heterochromia iridis but has no skin pigmentation or white forelocks. Interestingly, the proband's mother, who was not genotyped, showed heterochromia iridis but without hearing loss, skin pigmentation, or white forelocks. The phenotypic variability between the proband and his mother may be due to incomplete penetrance, highlighting the complex nature of genotype‐phenotype relationships and the potential influence of other genetic factors.

Exome sequencing has successfully resolved the etiology for HL in 40% (6/15) of the probands, consistent with previous global reports.^[^ ^24^, ^53^, ^54^, ^55^, ^56^, ^57^, ^58^ ^]^ In these studies, a single‐family to more than 1000 families were evaluated. Twenty‐three HL individuals from India were included in a multicentric study that screened 180 genes using the MiamiOtoGene panel, which resolved the genetic etiology in 26% (6/23) of cases.^[^ ^29^ ^]^

Six recessive genes (*LRGUK, DNAH9, ARMC4, DNAH2, RSPH6A*, and *ACE*) were implicated for MI. In these six genes, eight variants were identified including seven missense and one splice site variant. Of these, four variants were compound heterozygous. This cohort also identified two novel variants that have not been reported previously (Table 2).

*LRGUK* gene encodes a protein that is required for normal sperm assembly in multiple aspects such as acrosome attachment, manchette function, and the eventual initiation of the axoneme tail growth from the basal body. LRGUK‐1 (isoform 1) protein helps in the distal movement of the perinuclear ring to form an appropriate shape for the sperm head. Variants in *Lrguk‐1* in mice lead to reduced sperm concentration along with defects in sperm motility and morphology.^[^ ^59^ ^]^ Semen analysis of the proband and his brother in the DIS‐2 family indicated reduced sperm concentration (oligozoospermia) and motility (asthenozoospermia), suggesting the homozygous variant p.[Gly297Val] disrupted LRGUK protein function. Although numerous variants in this gene have been reported in mutational databases, to the best of our knowledge, this will be the first to report on the *LRGUK* gene variant associated with MI.

*DNAH9*, an axoneme dynein heavy chain gene, is localized to the distal compartment of the ciliary axonemes.^[^ ^60^ ^]^ *DNAH9* defects cause primary ciliary dyskinesia (PCD) leading to male infertility due to immotile cilia and dysfunctional sperm flagella. Semen analysis of proband DIS‐8 revealed sperm motility defects (asthenozoospermia) possibly due to a dynein heavy chain deficiency caused by two missense variants, p.[Gly469Ser] and p.[Arg1517Gln].

The *ARMC4* gene contributes to ciliary motility, cilium assembly, and microtubule‐based movement.^[^ ^61^ ^]^ Variants in this gene cause PCD, an autosomal recessive disorder characterized by ciliary structural anomalies, respiratory tract infections, and abnormal sperm motility.^[^ ^62^ ^]^ Although proband DIS‐9 has oligozoospermia (low sperm count), the presence of two novel variants, c.2097+1G>A and p.[Gly922Glu], suggests PCD. Further detailed clinical characterization is needed to correlate phenotype and genotype.

DNAH2, an axonemal inner arm dynein heavy chain (DHC) is a testicular specific protein essential for ciliary function.^[^ ^63^ ^]^ Variants in the *DNAH2* gene may lead to impaired sperm motility.^[^ ^64^ ^]^ Proband DIS‐12′s semen analysis revealed reduced motility and sperm tail defects possibly due to DNAH2 deficiency. Segregation analysis indicated that both parents were heterozygous for the variant p.[Arg827Trp] suggesting its recessive transmission resulting in MI in the proband.

The *RSPH6A* gene encodes a testes‐specific cilia‐associated protein^[^ ^65^ ^]^ crucial for stable spermatozoon flagella elongation. Mutant Rsph6a mice are infertile due to deformation in flagella and immotile spermatozoa.^[^ ^66^ ^]^ Genetic and semen analysis of proband DIS‐13 showed homozygous missense variant p.[Gly647Ser] and asthenozoospermia (reduced sperm motility), respectively. Two infertile brothers (II‐6 and II‐7), who could not be sampled, might also be homozygous for this variant; however, his fertile brother (II‐2) did not carry this variant (Figure 1g). This is the first study to associate this variant with male infertility.

The *ACE* gene encodes two isozymes: the somatic isozyme and testis‐specific isozyme. The former is expressed in several tissues including renal epithelial cells, vascular endothelial cells, and epididymis whereas the latter is expressed only in sperm.^[^ ^67^ ^]^ Ace knockout male mice showed reduced fertility due to the decreased capacity of the sperm to achieve fertilization in vivo. The absence of ACE isozymes leads to abnormalities in the transportation of sperm in the oviducts and affects its ability to bind to zona pellucida.^[^ ^68^ ^]^ Deletion polymorphism in the *ACE* gene in infertile men showed higher oxidative stress which is predicted to have a vital role in the pathogenesis of male fertility by its pro‐oxidative effect and is associated with atypical seminal variables.^[^ ^67^ ^]^ Proband DIS‐15 is homozygous for the missense variant p.[Arg971Gln] and his semen analysis revealed reduced sperm concentration (oligozoospermia).

Notably, genes like *LRGUK* and *RSPH6A* were previously implicated only in mice models, while the *ARMC4* gene was implicated in chronic destructive airway diseases due to primary ciliary dyskinesia. This study is the first to observe the effect of variants in these genes on male infertility phenotype. A detailed clinical characterization of the implicated families will provide more insights. Recently exome sequencing identified 17 variants in 12 reported and novel genes among a large cohort of infertile men in the Indian subpopulation.^[^ ^69^ ^]^

While over 2000 genes are believed to play a role in human spermatogenesis, only a small number have been implicated in monogenic inheritance.^[^ ^8^ ^]^ Since most of the reports have been on an outbred population, the possibility of identifying recessive genes is reduced. Genetic studies on infertile men pose challenges as the trait prevents perpetuation. For such studies, a large family is needed to localize a dominant gene, whereas consanguinity will be helpful to identify a recessive gene.^[^ ^70^ ^]^ This study identified six such recessive genes in six probands, four of whom were born to consanguineous parents. The remaining two non‐consanguineous cases each received the defective alleles from their parents. Implying a high carrier frequency in the general population. Testing a larger infertile cohort might identify other causative genes. However, non‐genetic factors could play a role in unresolved cases of infertility.

There are a few limitations in this study. First, due to a limited sample size, the analysis could be considered as a pilot report, and we could not conclusively estimate the frequency of DIS in our population. Ascertaining an adequate sample size poses a significant challenge due to the infrequent presence of both phenotypes (HL and infertility), which requires an extended duration of data collection. Secondly, the absence of copy number variants (CNV) analysis and functional assays of the variants. The causative variants identified were assessed using online in silico tools and segregation analysis. Thirdly, the lack of clinical data for certain cases due to lack of consent and unavailability.

Disorder heterogeneity impacts molecular diagnosis and presents a challenge.^[^ ^71^ ^]^ However, advancements in NGS technology have uncovered the molecular pathogenesis of complex disorders and facilitated therapeutic approaches. Its ability to analyze thousands of genes simultaneously makes it a powerful tool for identifying variants in particular rare variants.^[^ ^72^ ^]^

## 4 Conclusion

Prelingual genetic HL and MI can manifest as isolated traits or as part of a syndrome. While we initiated this study considering these conditions as either syndromic or linked, comprehensive screening has suggested that these phenotypes are more likely results of two independent events in over half of the cases. WES provides a cost‐effective approach to determine the genetic etiology of individuals presenting with HL and MI phenotypes. Understanding the exact cause of HL and MI aids in genetic prevention and facilitates genetic counseling for patients and their relatives. For idiopathic infertile men, this understanding can help avoid a drawn‐out trial‐and‐error approach in seeking various treatments in the hope of conception.

## 5 Experimental Section

### Subjects

This study was approved by the Institutional Human Ethical Committee (UM/IHEC/12‐2013‐I) and informed consent was obtained from each proband and their family members. Originally, a total of 115 families (Deaf X Deaf and Deaf X Normal) with prelingual hearing impairment were studied for a spectrum of deafness variants during the period 2010–2016. Out of the 103 male spouses with prelingual deafness, 14 were found to be infertile (13.5%). Additionally, an unmarried deaf proband with proven infertility was included in the study. This study focuses on these 15 unrelated probands and their families, originating from South India. Hearing was tested using pure tone audiometry which revealed severe to profound hearing loss. Semen analysis showed reduced volume and deformity in sperm motility and morphology establishing infertility. Initially, all these probands were assessed for *CATSPER2/STRC* contiguous deletions and *FOXI1* gene variants. However, none of them yielded positive results.^[^ ^73^ ^]^ Consequently, they were subjected to exome sequencing to identify the genes responsible for their hearing loss and/or infertility.

### Exome Sequencing

Exome sequencing (ES) was performed (MedGenome Labs Ltd., Bangalore, India) using Agilent SureSelect Human all exon V5 kit. Genomic DNA was sheared to produce 150–200 bp fragments. Library preparation was performed according to protocol. The library and probe sets were incubated at 65 °C for 16 h. The capture of resulting DNA‐RNA duplexes was performed by the addition of Myone streptavidin T1 beads (Invitrogen, USA). Exome Library QC was checked on a Bioanalyzer (Agilent, USA) and quantified using Qubit (Invitrogen, USA). Paired‐end sequencing was performed on Hi‐Seq2000 to obtain 2 × 75 bp reads for ES, using V3 Illumina by synthesis chemistry (Illumina, USA). FastQ data subjected to QC analysis were aligned to the reference genome GRCh37/hg19. Genome Analysis Toolkit^[^ ^74^ ^]^ was used for local read realignment around indels with the GATK Best Practice Variant Detection v3 recommendations. Gene annotation of the variants was performed using VariMAT v2.3.8, HGMD v2016, and MitoMAP v08 Aug 2016 program against the Ensembl release 84 human gene model.

### Variant Analysis

Genes associated with HL and MI (Tables S1 and S2, Supporting Information) were prioritized using pathway analysis online tools. Common variants were filtered out based on allele frequency (>1%) in 1000 Genome Project, gnomAD, Exome Sequencing Project, and an internal Indian population database (MedGenome). Clinically relevant variants were annotated using a set of databases – ClinVar (https://www.ncbi.nlm.nih.gov/clinvar/), OMIM (https://www.omim.org/), GWAS (https://www.ebi.ac.uk/gwas/) and HGMD (http://www.hgmd.cf.ac.uk/ac/index.php). All presumably non‐disturbing protein variants (introns, UTRs, intergenic, synonymous, etc.) were filtered out. Only nonsynonymous and splice‐site variants were used for clinical interpretation. HL‐associated gene variations were classified using American College of Medical Genetics and Genomics (ACMG) guidelines specific to hearing loss.^[^ ^75^ ^]^ Variants in genes associated with MI were classified using ACMG guidelines.^[^ ^76^ ^]^ The final list of variants was manually reviewed using IGV.^[^ ^77^ ^]^ In silico pathogenicity prediction tools such as PolyPhen2,^[^ ^78^ ^]^ SIFT,^[^ ^79^ ^]^ Mutation Taster^[^ ^80^ ^]^ were used to evaluate the possible impact of missense variants on protein function. ConSurf was used to compute evolutionary conservation.^[^ ^81^ ^]^ All pathogenic variants were confirmed using Sanger sequencing; segregation analysis was performed using appropriate exon‐specific primers (Table S3, Supporting Information). Supporting Information and Figure S1 (Supporting Information) show the detailed methodology adopted for filtering variants detected by WES.

## Conflict of Interest

The authors declare no conflict of interest.

## Author Contributions

J.J.M. collected the data, performed the experiments and analysis, wrote the original draft, reviewed, and revised the manuscript. C.J. collected the data and performed the analysis, reviewed and revised the manuscript. P.A. collected the data, reviewed, and revised the manuscript. H.A. performed the analysis, reviewed, and revised the manuscript. C.R.S.S. conceived and designed analysis, reviewed and revised the mansucript.

## Supporting information

Supporting Information

## Data Availability

The data that support the findings of this study are openly available in NCBI ClinVar at https://www.ncbi.nlm.nih.gov/clinvar, reference number 505964.
