# Supplementary material for: Unraveling the Genetic Basis of Combined Deafness and Male Infertility Phenotypes through High‐Throughput Sequencing in a Unique Cohort from South India
Source: Adv Genet (Hoboken). 2024 May 8;5(2):2300206. doi: 10.1002/ggn2.202300206 (PMC11170077; doi:10.1002/ggn2.202300206)
Supplement: Supplementary file 1 — Supporting Information [file GGN2-5-2300206-s001.docx]

**Supplementary Data**

**Exome Sequencing and Data Analysis**

**Library Preparation**

Genomic DNA was sheared to produce 150–200 bp fragments. Following size selection, end-repair, phosphorylation, and A-tailing adapters were ligated to the DNA fragments according to the manufacturer's protocol (Agilent SureSelect Human all exon V5 kit). Hybridization was carried out using 700 ng of library and 5μL of biotin-labeled RNA probe sets (Agilent), designed specifically for the desired targets of the whole exome panel.

The library and probe sets were incubated at 65°C for 16 hours. Capture of resulting DNA-RNA duplexes was performed by the addition of Myone streptavidin T1 beads (Invitrogen, USA). Multiple washes at high stringency were used to remove off-target material and any nonhybridized fragments. Indexed primers and Herculase II Fusion DNA Polymerase (Agilent, USA) were used for the amplification of specific libraries. Exome Library QC was checked on a Bioanalyzer (Agilent, USA) and quantified using Qubit (Invitrogen, USA). The libraries were diluted to 10 nM concentration in the TE buffer.

The libraries were denatured and further diluted to 8.5 pM concentrations using the HT buffer to be loaded for cluster generation in the cBOT (Illumina, USA). Cluster amplification of denatured templates was performed according to the manufacturer's protocol (Illumina, USA) using V3 Chemistry and V3 Flow cells. Paired-end sequencing was performed on Hi-Seq2000 to obtain 2 × 75 bp reads for whole exome sequencing, using V3 Illumina sequencing by synthesis chemistry (Illumina, USA).

**Data processing and variant calling**

FastQ data were subjected to QC analysis and high-quality reads were filtered using fastx_toolkit ((http://hannonlab.cshl.edu/fastx_toolkit/) and FastQC tool (http://www.bioinformatics. babraham.ac.uk/projects/fastqc/). They were aligned to the reference genome GRCh37/hg19 using the BWA-MEM tool v bwa-0.7.12 and a SAM file was generated. Binary format (BAM) file was converted from SAM files and was sorted and indexed using SAM tools (v.samtools-1.2) (Li et al, 2009).

The aligned reads were sorted by Picard (v.picard-tools-1.140) to remove PCR duplicates (http://picard.sourceforge.net). Genome Analysis Toolkit v3.6-0-g89b7209 (GATK; http://www.broadinstitute.org/gatk) (DePristo et al, 2011) was used for local read realignment around indels with the GATK Best Practice Variant Detection v3 recommendations (http://www.broadinstitute.org/gatk/guide/topic? name=bestpractices). A clean BAM file was produced using the base quality score recalibration in the BAM file. GATK v3.6 (DePristo et al, 2011) and SAM tools v1.2 (Li et al, 2009) were used for variant calling in the cleaned BAM files. VCF files were then created for all the probands tested.

**Confirmation of variants**

The candidate variants identified in the DIS samples using WES were confirmed using conventional Sanger sequencing. The primers for appropriate genes and exons were designed using Primer3 online tool (Table S3). All the amplified PCR products were purified using QIAquick® PCR purification kit (Qiagen, Valencia, CA, USA) and sequenced using ABI Prism Big-Dye Terminator 3.1 cycle sequencing reaction kit on an ABI 3730 automated sequencer (Applied Biosystems, Foster City, USA). The chromatogram sequences obtained were compared with an appropriate gene sequence in National Center for Biotechnology Information (NCBI:http://www.ncbi.nlm.nih.gov/) and UCSC genome Browser (https://genome.ucsc.edu/) to identify the nucleotide base-pair changes. Further, these variants were screened in their available family members for segregation analysis.

***Illumina HiSeq***

***DVD – Deafness Variation Database***

***VUS-Variant of Uncertain Significance***

***IGV- Integrated Genome Viewer***

***Yes***

***No***

**High Impact Variants**

**Moderate Impact Variants**

**Low Impact Variants**

**ACMG Guidelines 2015 Classification**

**Pathogenic**

**Likely Pathogenic**

**VUS**

**Benign**

**Likely Benign**

**Manual Review in IGV**

**Sanger Validation**

**Segregation Analysis and Clinical Correlation**

**Causative Gene/variant**

***Variant Classification***

***Deafness and/or Male Infertility Genes***

**Whole Exome Sequencing
(DIS n=15)**

**FastQ Raw Data
(QC Analysis)**

**Aligned to reference genome
(GRCh37/hg19)**

**Variant calling (GATK tool)**

**Variant Annotation**

**(GRCh37/hg19)**

**Filtering SNPs – MAF>1-2%**

**(1000G, ExAC, EVS, dbSNP 147)**

**Gene/variant prioritization**

(DVD, OMIM, HGMD, ClinVar, SwissVar)

***Filtered out***

**Figure S1:** Flowchart illustrating the filtering schema for exome data analysis.

**Table S1:** List of 233 auditory genes prioritized during exome data analysis.

| ACTB | ACTG1 | ADCY1 | ADGRV1 | AIFM1 |
| --- | --- | --- | --- | --- |
| ALMS1 | ATP2B2 | ATP6V1B1 | BCAP31 | BCS1L |
| BDP1 | BSND | BTD | C10orf2 | CABP2 |
| CACNA1D | CATSPER2 | CCDC50 | CD151 | CD164 |
| CDC14A | CDH23 | CEACAM16 | CHD7 | CIB2 |
| CISD2 | CLDN14 | CLIC5 | CLNN9 | CLPP |
| CLRN1 | COCH | COL11A1 | COL11A2 | COL11A23 |
| COL2A1 | COL4A3 | COL4A4 | COL4A5 | COL4A6 |
| COL9A1 | COL9A2 | COL9A3 | CRYL1 | CRYM |
| CYRM | DCDC2 | DDC2 | DFNA5 | DFNB31 |
| DFNB59 | DIABLO | DIAPH1 | DIAPH3 | DMXL2 |
| DNMT1 | DSPP | EDN3 | EDNRB | ELMOD3 |
| EPE8L2 | EPS8 | EPS8L2 | ERAL1 | ESPN |
| ESPS8 | ESRP1 | ESRRB | EYA1 | EYA4 |
| FAM189A2 | FAM65B | FGF3 | FGFR1 | FGFR2 |
| FOX1 | FOXI1 | GAB1 | GATA3 | GIPC3 |
| GJB1 | GJB2 | GJB21 | GJB3 | GJB4 |
| GJB6 | GPR98 | GPSM2 | GRAP | GRHL2 |
| GRXCR1 | GRXCR2 | GSDME | HARS | HARS2 |
| HGF | HOMER2 | HSD17B4 | IFNLR1 | ILDR1 |
| JAG1 | KARS | KCNE1 | KCNJ10 | KCNQ1 |
| KCNQ4 | KITLG | LARS2 | LHFPL5 | LHX3 |
| LMX1A | LOXHD1 | LOXL3 | LRTOMT | LRTOMT/COMT2 |
| LRTOMTMYO7A | MARVELD2 | MARVELD2/BDP1 | MCM2 | MET |
| METTL13 | MIR96 | MIRN96 | MITF | MPZL2 |
| MSRB2 | MSRB3 | MTO15A | MTRNR1 | MTTL1 |
| MTTS1 | MT-TS1 | MYH14 | MYH9 | MYO15A |
| MYO1A | MYO1C | MYO1F | MYO3A | MYO6 |
| MYO67 | MYO7A | MYO7A2 | NAR52 | NARS2 |
| NDP | NF2 | NLRP3 | OPA1 | OSBPL2 |
| OTOA | OTOA pseudogene | OTOF | OTOG | OTOGL |
| P2RX2 | PAX3 | PCDH15 | PDE1C | PDZD7 |
| PEX1 | PEX6 | PEX7 | PHYH | PJVK |
| PLS1 | PNPT1 | POLR1C | POLR1D | POU3F4 |
| POU4F3 | PPIP5K2 | PRPS1 | PTNPT1 | PTPRQ |
| RDX | REST | ROR1 | RPGR | S1PR2 |
| SDHD | SEMA3E | SERAC1 | SERPINB6 | SIPR2 |
| SIX1 | SIX5 | SLC12A2 | SLC17A8 | SLC22A4 |
| SLC26A4 | SLC26A5 | SLC4A11 | SLITRK6 | SMAC/DIABLO |
| SMPX | SNAI2 | SOX10 | SOX2 | SPSNS2 |
| STRC | STRC pseudogene | SYNE4 | TBC1D24 | TBC1D245 |
| TBX1 | TCOF1 | TECTA | TECTA5 | TECTA6 |
| TECTB | TFCP2L3 | TIMM8A | TJP2 | TMC1 |
| TMC14 | TMEM132E | TMIE | TMPRSS3 | TMPRSS5 |
| TNC | TPRN | TRIOBP | TRRAP | TSPEAR |
| TWNK | USH1C | USH1G | USH2A | VLGR1 |
| WBP2 | WFS1 | WHRN |  |  |

**Table S2:** List of 180 male infertility genes prioritized during exome data analysis.

| ACSBG2 | ADAMTS20 | ADCY10 | AKAP3 | AKAP4 |
| --- | --- | --- | --- | --- |
| ALF | APOB | AR | ART3 | ATM |
| ATMAC | AURKC | BEX2/1 | BOULE | BPY2 |
| BRCA2 | CACNA1C | CATSPER1 | CATSPER2 | CATSPER3 |
| CATSPER4 | CDY | CDY1 | CFTR | COX10 |
| CREM | CSNK2A2 | CYP19A1 | CYP1A1 | DAZ |
| DAZL | DBY | DDX25 | DDX3Y | DHAH5 |
| DNAH11 | DNAH5 | DNAI1 | DNEL1 | DNMT3 |
| DNMT3B | DNMT3L | DPY19L2 | DRFFY | EDDM3A |
| EDDM3B | EGF | ERCC1 | ERCC2 | ESR1 |
| ESR2 | FAS | FASL | FASLG | FHL5 |
| FKBP6 | FKBPL | GAMT | GAPDHS | GNA12 |
| GOLGA8C | GOPC | GSTM1 | GSTP1 | GSTT1 |
| GTF2A1L | H1FNT | H2BFWT | HFE | HIWI2/3 |
| HLADQA1 | HLA-DRB1 | HNRNPC | HRB | HSFY |
| HSFY1 | HSP90 | IL1A | IL1B | JUN |
| JUND | KIT | KITLG | KLHL10 | LAP3 |
| LRGUK | MAATS1 | MAK | MBOAT1 | MEI1 |
| MEST | MLH1 | MLH3 | MS | MSH4 |
| MTHFR | MTR | MTRR | NALP14 | NANOS2 |
| NANOS3 | NLRP14 | NPHP1 | NR5A1 | NRB0B1 |
| NRG1 | NRIP1 | OAZ3 | PBK | PDYN |
| PICK1 | PIGA | PLA2G6 | PKD1L1 | POIA3 |
| POLG | PON1/2 | POTE B | PPM1G | PRDM16 |
| PRDM9 | PRKAR1A | PRM1 | PRM2 | PRY |
| PUM2 | PVRL2 | RBMX | RBMY1A1 | RBMY1F |
| RID2 | RNF141 | SABP | SCA1 | SDHA |
| SHBG | SLC6A8 | SLC9A10 | SOHLH1 | SOX8 |
| SP1 | SPAG16 | SPATA | SPATA12 | SPATA16 |
| SPATA17 | SPATA4 | SPATA42 | SPATA6 | SPATA8 |
| STRA8 | SUN5 | SYCP1 | SYCP3 | TAF7L |
| TEKT1 | TEKT2 | TEX101 | TGIF2LX | TNFR1 |
| TNFR2 | TPN1 | TPN2 | TSPY | TSSK2 |
| TSSK4 | TSSK6 | TWF1 | TXNDC3 | UBE2B |
| USP26 | USP9Y | UTP14C | UTY | VASA |
| XPC | XPD | XRCC1 | YBX2 | ZNF230 |

**Table S3:** Details of the primer sequences used for amplification of candidate genes for hearing loss and male infertility identified in exome sequencing.

| **S. No** | **Gene** | **Exon /Intron** | **Primer Sequence (forward and reverse)** | **Product size (bp)** | **Annealing Temp (^o^C)** |
| --- | --- | --- | --- | --- | --- |
| 1. | *TRIOBP* | 7 | ACAGAACCACCCAACAAGAG | 412 | 59 |
|  |  |  | GTACAAGTGGGTCTGAGGTTATC |  |  |
| 2. | *LRGUK* | 7 | GAGGGAAGGACAAGAATGCCT | 301 | 59 |
|  |  |  | CCAAGACATGTGAAGTGCAACA |  |  |
| 3. | *SLC26A4* | 3 | GCAAATTGGTTGTGACTGAG | 294 | 62 |
|  |  |  | GAAGGGTAAGCAACCATCTGTCAC |  |  |
| 4. | *DNAH9* | 7 | ATTTCCTAAGCAACCACCGC | 643 | 59 |
|  |  |  | CCCTACAGATGAGAGGCATCC |  |  |
|  |  | 20 | TATGGACCAGGACACCACCC | 506 | 60 |
|  |  |  | GAGCCTAGACAATGCAGGAACT |  |  |
| 5. | *ARMC4* | 14 | ACTACCGGGCTGCAATCAAA | 206 | 59 |
|  |  |  | CCTGAGTCCCATTCTCACAGA |  |  |
|  |  | 18 | GCCAAAGATGTACCTCACAGC | 389 | 58 |
|  |  |  | GAATCCAGGTTTCTGGACTGCAA |  |  |
| 6. | *SOX10* | 5 | AGCCCTCAGGACCCTATTAT | 425 | 60 |
|  |  |  | GCAACAGTCAACCTCCTTCT |  |  |
| 7. | *DNAH2* | 16 | CAAGAATGATGGTCCTGAGGTAG | 617 | 59 |
|  |  |  | GGTCTGAAGGCCATAGGATAAAG |  |  |
| 8. | *RSPH6A* | 6 | GGGCCTTTGAGGGAGAATTT | 311 | 61 |
|  |  |  | GCTTGGGGAAAGTGGCTAGA |  |  |
| 9. | *COL4A3* | 6 | TTTCCCTTGGGTTCAGTGCT | 219 | 59 |
|  |  |  | GGAAGCCTGTCTGCCTTTCAT |  |  |
| 10. | *ACE* | 19 | CTTTCCTCTCTCTGCCGTCC | 538 | 60 |
|  |  |  | TGGACCCTTTGCTTGGTTCA |  |  |

**
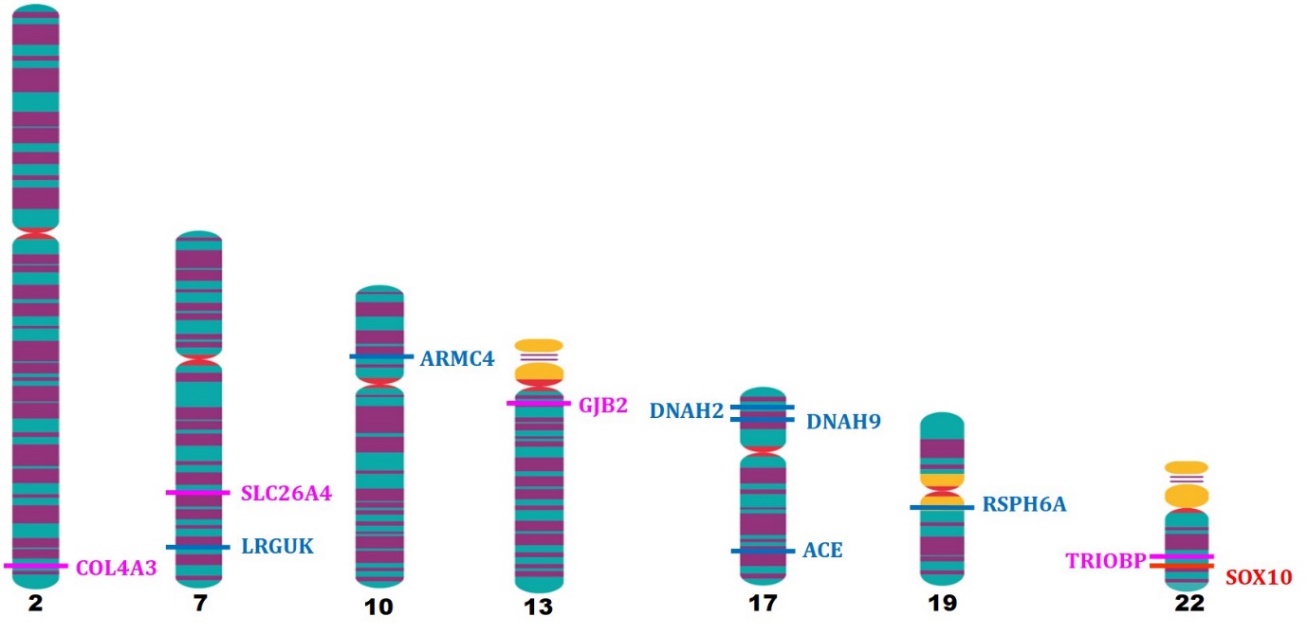
**

**Figure S2:** Eleven candidate genes mapped on human chromosomes to recessive or dominant hearing loss or male infertility were identified using ES in the present study. The four hearing loss recessive genes are shown in purple and one dominant gene is in red. Six male infertility genes are shown in blue.


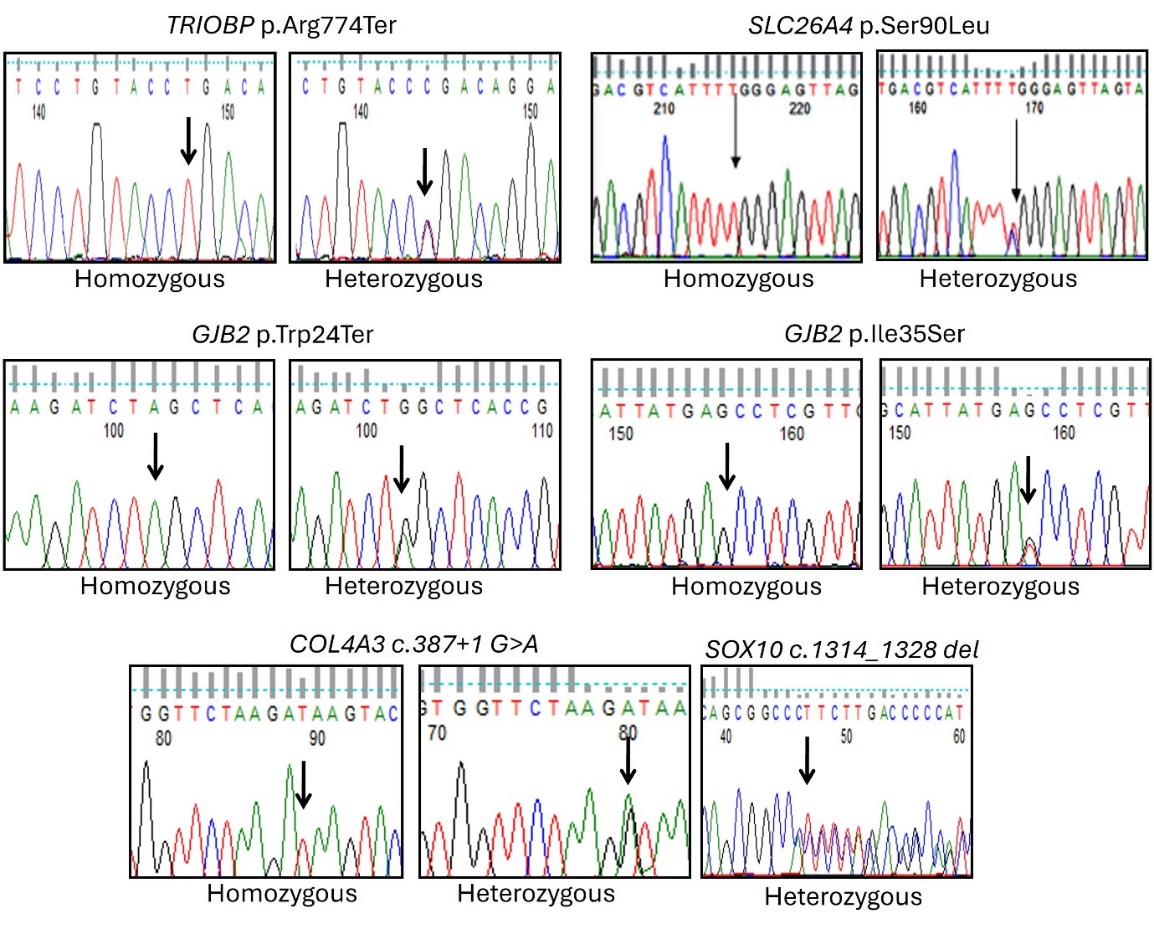


**Figure S3**: Chromatograms of the causative hearing loss gene variants identified in the exome sequencing.


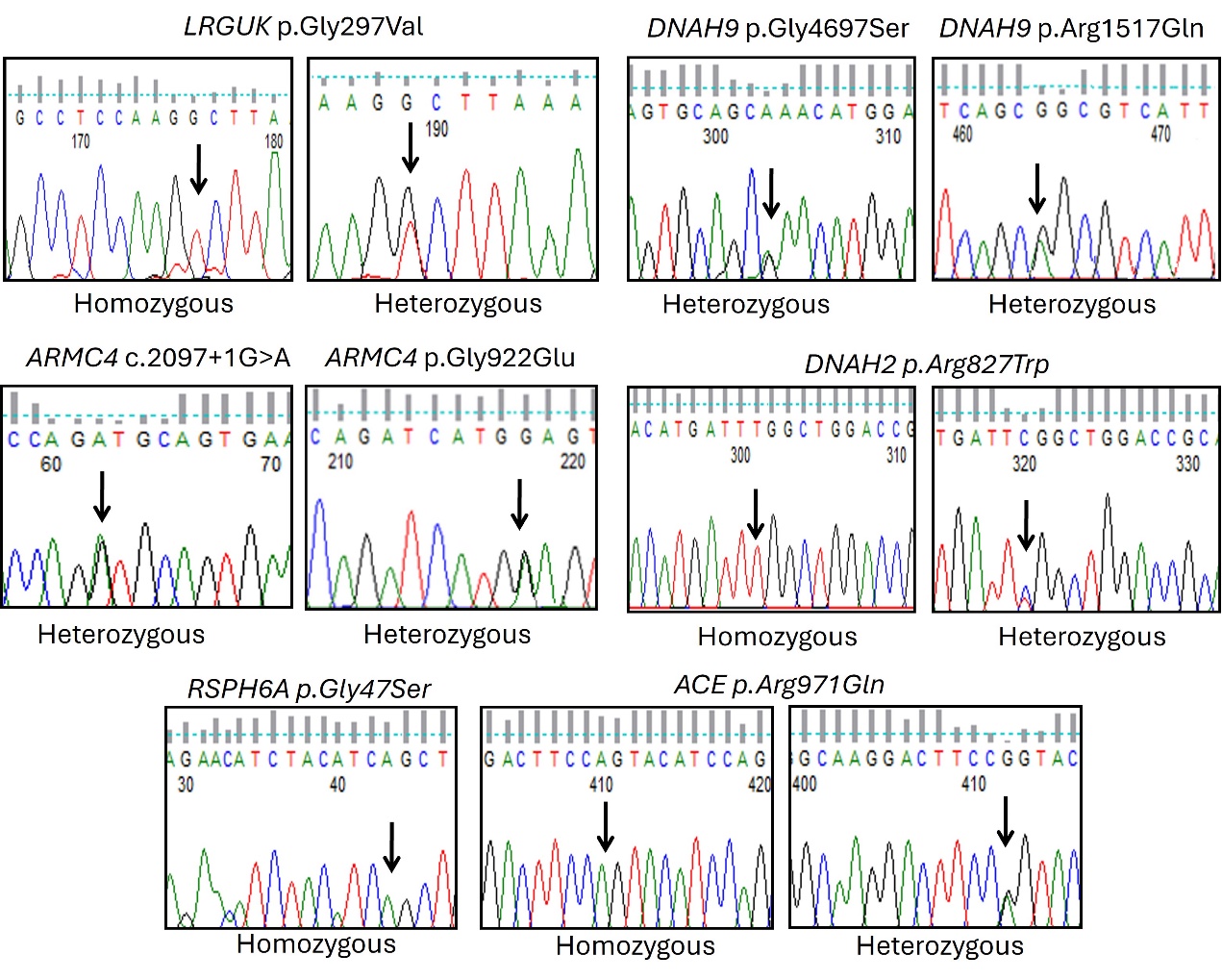


**Figure S4**: Chromatograms of the causative male infertility gene variants identified in the exome sequencing.


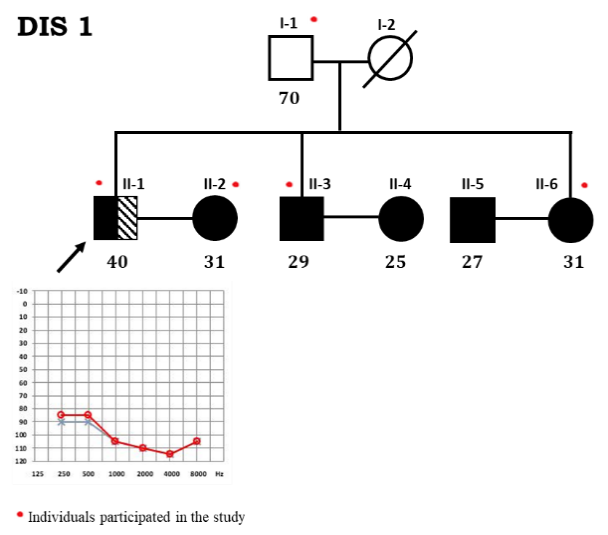

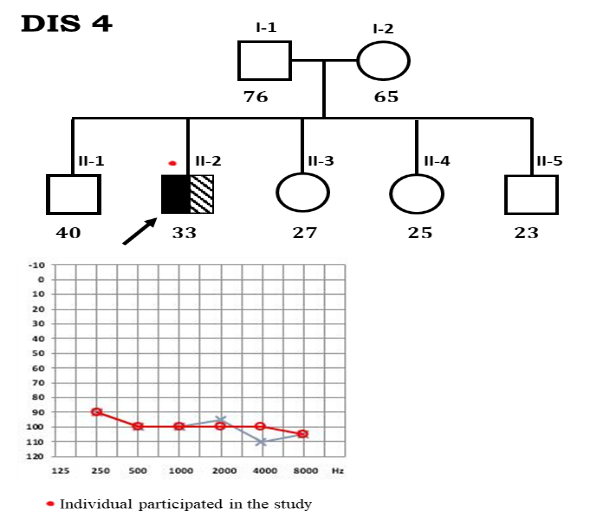


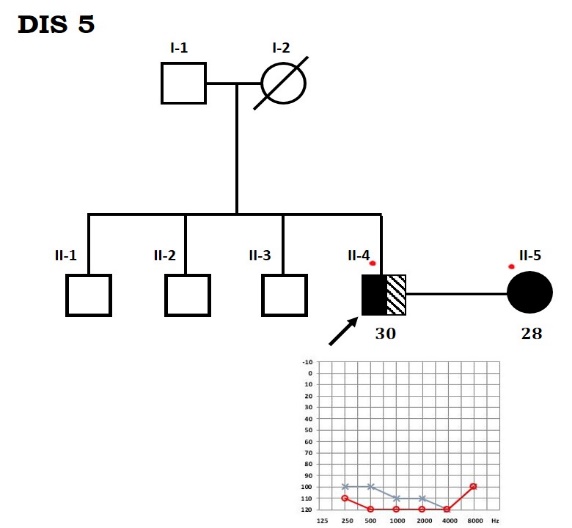

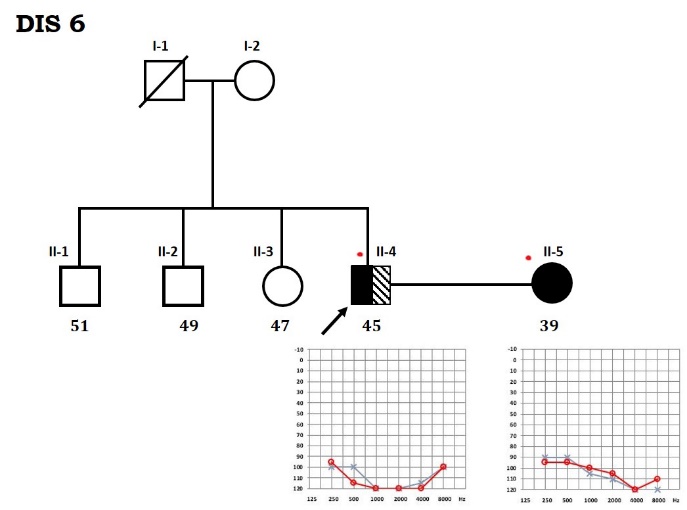


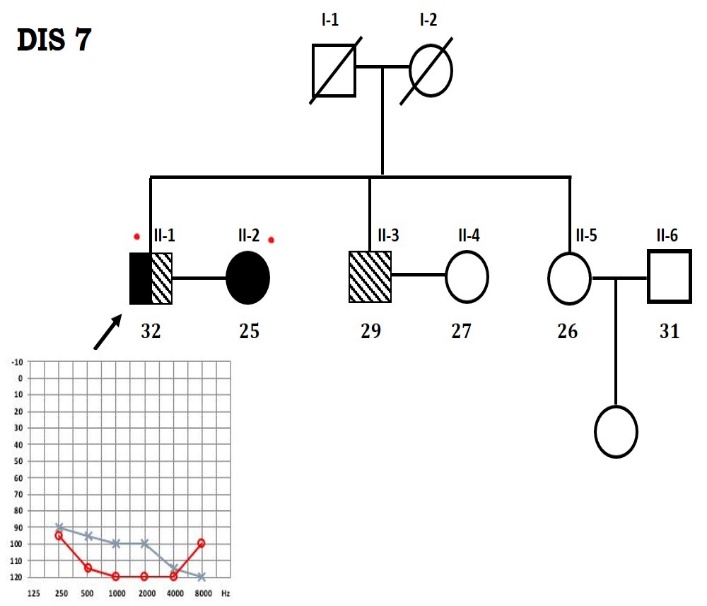

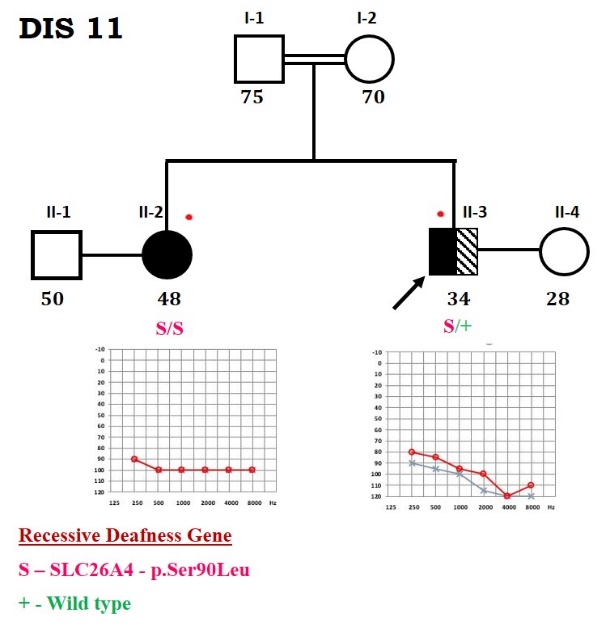


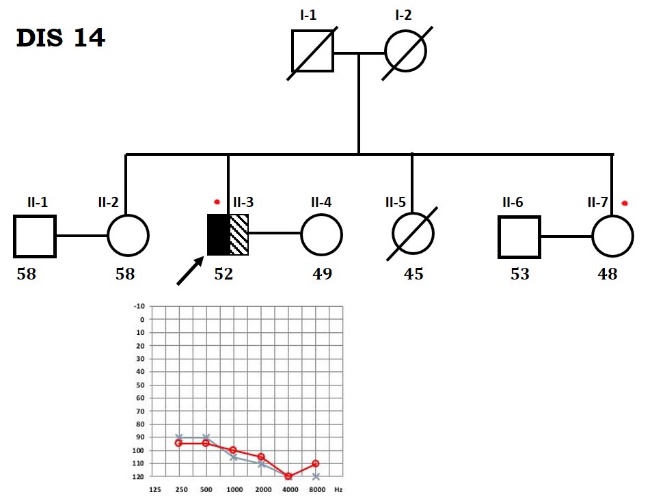


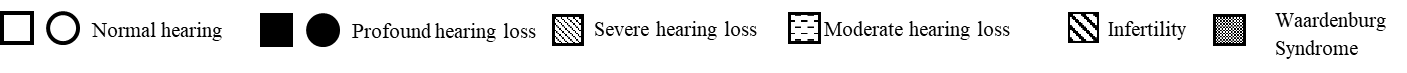


**Figure S5:** Families unresolved for HL and/or MI etiology.
